# Syncopedia: training a new generation of syncope specialists

**DOI:** 10.1007/s10286-017-0481-z

**Published:** 2017-11-14

**Authors:** Jelle S. Y. de Jong, Frederik J. de Lange, Nynke van Dijk, Roland D. Thijs, Wouter Wieling, on behalf of the Syncopedia editorial board

**Affiliations:** 1 Departments of Cardiology, Academic Medical Centre, University of Amsterdam, Amsterdam, The Netherlands; 2 General Practice/Family Medicine, Academic Medical Centre, University of Amsterdam, Amsterdam, The Netherlands; 3 Department of Neurology, Leiden University Medical Centre, Leiden, The Netherlands; 4 SEIN—Stichting Epilepsie Instellingen Nederland, Heemstede, The Netherlands; 5 Internal Medicine, Academic Medical Centre, University of Amsterdam, Amsterdam, The Netherlands

## What is syncopedia?

**Fig. 1:**
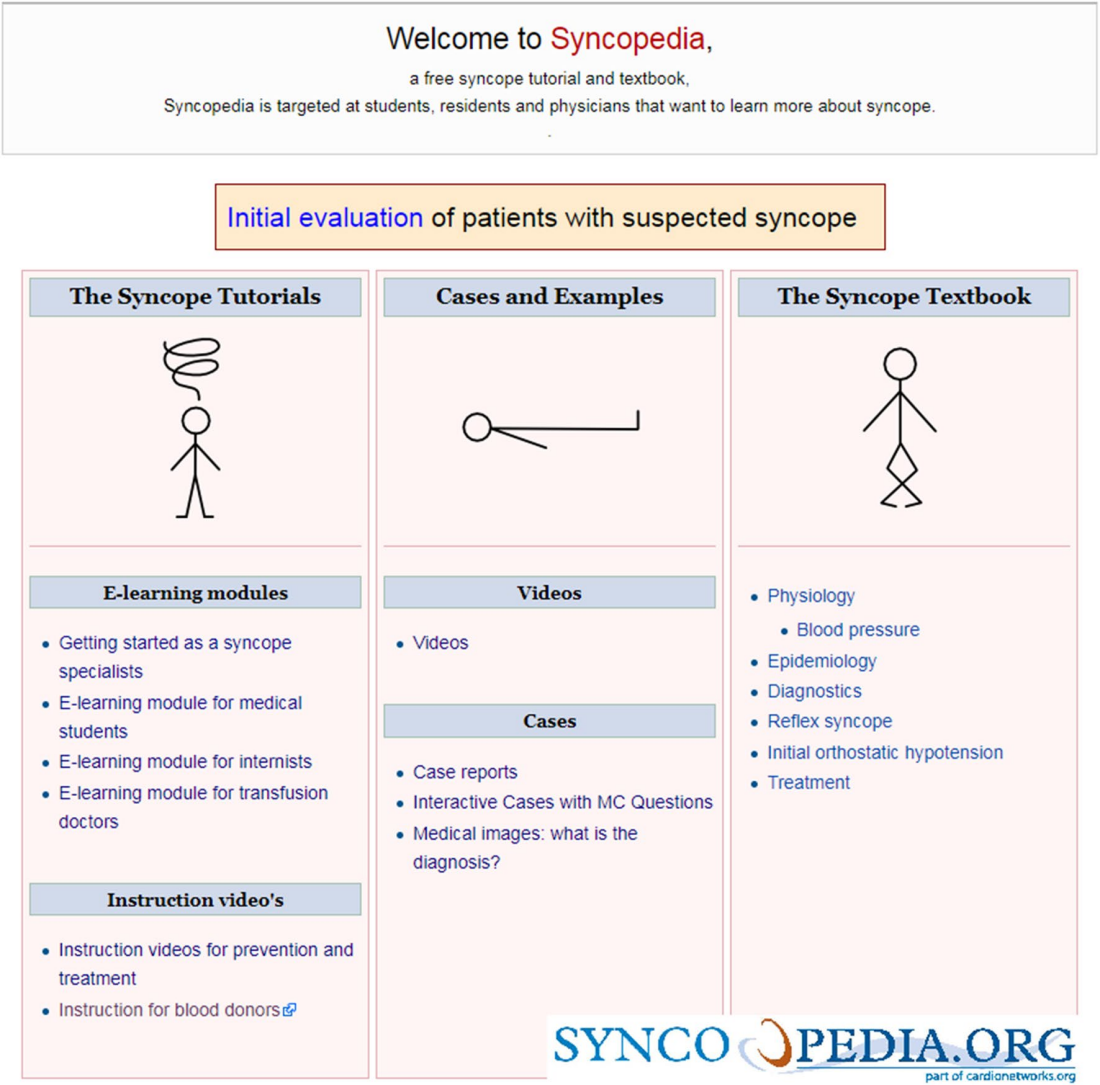
Initial evaluation of patients with suspected syncope

Syncopedia is a free-access educational website targeted at students, residents and physicians who want to learn about syncope (Fig. 1). The website is an initiative of the Syncopedia Foundation, a nonprofit organization founded in 2014. The goal of the Syncopedia Foundation is: “improving medical knowledge, especially in the field of syncope, and providing access to this knowledge by facilitating publications in digital or other forms, for example by building and maintaining websites.” The goal of the Syncopedia website is to enhance physicians’ knowledge of (suspected) syncope and reduce misdiagnosis, unnecessary testing, and excessive specialist consultations.

Syncope is a symptom with many possible causes, requiring all-round rather than organ-specific knowledge. Unfortunately, thorough history taking and a knowledge of cardiovascular physiology are no longer included in the core medical curricula [1, 2].

In this editorial, we address the importance of history taking in patients with suspected syncope and emphasize that, while a knowledge of cardiovascular physiology is important, a deep understanding is better for optimal syncope care.

## Initial evaluation of patients with transient loss of consciousness

To start with, we must define a transient loss of consciousness (T-LOC) [3]. T-LOC is a real or apparent loss of consciousness for a short duration, as characterized by (1) amnesia for the period of unconsciousness, (2) abnormal motor control, and (3) loss of responsiveness. T-LOC is extremely common and caused by many disorders ranging from the benign to the lethal, and treated by different disciplines. This necessitates an efficient diagnostic work-up.

The European Society of Cardiology Guideline on Syncope recommends that the initial work-up of T-LOC consists of history taking, a physical examination, and an ECG. The emphasis on history taking is justified by its high diagnostic yield [3]. A “highly likely” diagnosis can be made by a non-expert during the initial evaluation in about 60% of patients. Expert history taking that focuses on the narrative in order to elucidate predisposing factors and physiological triggers that can elicit T-LOC can boost the diagnostic yield to 90% [4].

T-LOC has never been claimed by any specialty, so it has become an “orphan” condition that falls in between disciplines. As a result, it is not optimally taught in the specialty training programs [5, 6]. Specialists fall back on attempts to rule out causes in their own field. This involves applying tests with a low diagnostic yield aimed merely at ruling out rather than ruling in diagnoses, resulting in excessive visits to specialists, redundant testing, and high costs [4, 5]. While it is critical that causes of T-LOC with serious prognostic implications are ruled out, this is not of great value for the patient who simply wants an explanation and receive treatment. They are not interested in a “you do not have” approach [4].

## Physiological reasoning: non-invasive continuous monitoring of finger arterial pressure

Due to rapid advances in technology, molecular biology, genetics, clinical epidemiology, and evidence-based medicine, as well as the wide institution of electronic health records, the interest in basal bedside medicine and clinical physiology has decreased. Young doctors are more likely to diagnose patients using a monitor to see laboratory results and radiological images instead of practicing bedside medicine and building a comprehensive history by asking questions and applying physiological reasoning [1, 7, 8]. However, it is important to note that pathophysiology is the platform on which modern medicine is built; it often plays a decisive role in the diagnosis and treatment of syncope.

As reflex syncope and orthostatic hypotension, the most common causes of syncope, are related to abnormal control of arterial blood pressure, physicians caring for patients with suspected reflex syncope or orthostatic hypotension should have an in-depth understanding of circulatory physiology and pathophysiology. The clinician and scientist Sharpey-Schafer was the first to couple clinical observations of provoked syncope to continuous intra-arterial blood pressure monitoring and cardiac output measurements. His clinical observations and astute clinical reasoning were fundamental [8].

Today, doctors interested in syncope benefit from the availability of continuous noninvasive measurement of finger arterial pressure (FinAP) and pulse wave analysis for studying the hemodynamics underlying syncope [9]. However, the knowledge of integrative cardiovascular physiology required to interpret the results of the new technologies is no longer taught in the medical curriculum, and information that syncope doctors need for their training is not available in an easy format [2]. This understanding of cardiovascular physiology is a prerequisite for the analysis of FinAP tracings.

## Training a new generation of syncope specialists

A syncope specialist is a physician with a sufficient knowledge of historical clues and physical findings to recognize major causes of T-LOC (including mimics) and syndromes of orthostatic intolerance [6]. The physician most likely to see a patient with suspected syncope is a general internist, neurologist, cardiologist, or geriatrician. Syncope specialists are often cardiologists with an interest in electrophysiology and pacing, neurologists with a special interest in autonomics and epilepsy, or internists with an interest in cardiovascular physiology. However, the specialty training programs do not thoroughly cover the physiology and historical clues needed to recognize major causes of T-LOC.

Using Syncopedia, we are trying address these knowledge gaps. The scheme at the top of the website entitled “Initial evaluation of patients with suspected syncope” is a diagnostic algorithm that can be used in emergency departments (EDs) to identify or exclude causes of T-LOC that may have serious prognostic implications.

Diagnosing the underlying cause of an episode of T-LOC is considered less important in the frenetic emergency environment, which is characterized by a “do-it-faster, do-it-standardized, multitask” approach with constant interruptions [4, 5]. Patients in whom a dangerous underlying pathology is highly unlikely are often diagnosed with a “common faint” or “orthostatic hypotension” and sent home or advised to see their GP without further instructions.

The educational material, consisting of syncope tutorials, cases and examples, and the syncope textbook, is intended to enhance the basic knowledge of medical students, residents, and doctors and to train a new generation of syncope specialists to handle these patients in a variety of clinical settings.

## Work in progress

Syncopedia is a work in progress; all the information necessary to learn about suspected syncope will become available over time. If you think that important information is missing, or you would like information on a specific subject that is not yet covered, please let us know using the forum on www.syncopedia.org or by contacting the corresponding author.

## Appendix

This manuscript was written on behalf of the Syncopedia editorial board.

W Wieling, Academic Medical Centre, Amsterdam, The Netherlands (Editor-in-chief).

JSY de Jong, Academic Medical Centre, The Netherlands (Webmaster).

J Butler, Christchurch Hospital, Christchurch, New Zealand.

N van Dijk, Academic Medical Centre, Amsterdam, The Netherlands.

R Freeman, Harvard Medical School, Boston, USA.

FJ de Lange, Academic Medical Centre, Amsterdam, The Netherlands.

JWM Lenders, Radboud University Nijmegen Medical Center, Nijmegen, The Netherland and Technische Universität Dresden, Germany.

RD Thijs, Stichting Epilepsie Instellingen Nederland, Heemstede, The Netherlands.

AS Vink, Academic Medical Centre, The Netherlands.

MH Harms, University Medical Centre Groningen, Groningen, The Netherlands.
